# Glucose to lactate shift reprograms CDK-dependent mitotic decisions and its communication with MAPK Sty1 in *Schizosaccharomyces pombe*

**DOI:** 10.1242/bio.060145

**Published:** 2023-10-24

**Authors:** Priyanka Sarkar, Susmita Misra, Agamani Ghosal, Soumyajit Mukherjee, Alok Ghosh, Geetanjali Sundaram

**Affiliations:** Department of Biochemistry, University of Calcutta, Kolkata 700019, India

**Keywords:** Cdc2, Sty1, *S. pombe*, Lactate, Mitosis, Metabolism

## Abstract

Cell cycle regulation in response to biochemical cues is a fundamental event associated with many diseases. The regulation of such responses in complex metabolic environments is poorly understood. This study reveals unknown aspects of the metabolic regulation of cell division in *Schizosaccharomyces pombe*. We show that changing the carbon source from glucose to lactic acid alters the functions of the cyclin-dependent kinase (CDK) Cdc2 and mitogen-activated protein kinase (MAPK) Sty1, leading to unanticipated outcomes in the behavior and fate of such cells. Functional communication of Cdc2 with Sty1 is known to be an integral part of the cellular response to aberrant Cdc2 activity in *S. pombe*. Our results show that cross-talk between Cdc2 and Sty1, and the consequent Sty1-dependent regulation of Cdc2 activity, appears to be compromised and the relationship between Cdc2 activity and mitotic timing is also reversed in the presence of lactate. We also show that the biochemical status of cells under these conditions is an important determinant of the altered molecular functions mentioned above as well as the altered behavior of these cells.

## Teaser

**Summary:** The metabolic status of *Schizosaccharomyces pombe* cells dictates the regulation of cell division decisions and the influence of the MAPK pathway on mitotic timing.

## INTRODUCTION

Cell division is a metabolically taxing process requiring the production of large amounts of biomass and energy. Therefore, a cell's decision to initiate division is influenced both by its metabolic status and the availability of nutrients (Kalucka et al., 2015). The relationship between cellular metabolism and the cell cycle machinery is bidirectional. The ability of a cell to enter the cell cycle depends on the availability of metabolites. Conversely, the cell cycle machinery commits to regulate metabolic networks in order to support cell survival and proliferation (Kaplon et al., 2015). The mechanism of the combined regulation of metabolic events and components of the cell cycle is not well understood and investigation of the same is very important. Their reciprocal regulation plays a pivotal role not only in regulating mitotic commitment and cell cycle progression but also to adjust metabolic pathways in response to both intracellular and external cues.

There have been reports in recent years demonstrating that metabolism and cell cycle regulation are strongly connected. It has been shown that cell division is accompanied by metabolic oscillations in both yeast and mammalian cells, indicating that these connections are conserved (Kaplon et al., 2015; Liu et al., 2021). It has long been known that biosynthetic processes exhibit temporal changes in a cell cycle-dependent manner (Hartwell et al., 1974; Jong et al., 1984; Maitra and Lobo, 1977; Game, 1976; Dickinson and Williams, 1987; Wrobel et al., 1999; Cho et al., 1998; Spellman et al., 1998), which leads to the dynamic metabolic behavior that is a driving force for the cell cycle (Zylstra and Heinemann, 2022; Papagiannakis et al., 2017). Cross-talk between cell cycle regulation and carbohydrate metabolism has also been reported **(**Tudzarova, 2011; Colombo, 2010; Bao, 2013). In fact, several glycolytic enzymes such as 6-phosphofructo-2-kinase/fructose-2, 6-bisphosphatase3 (PFKFB3), hexokinase 2 (HK2), phosphofructokinase, platelet type (PFKP) and pyruvate kinase M2 (PKM2) have been reported to be regulated in a cell cycle-dependent manner (Jennifer et al., 2007; Herrero-Mendez, 2009; Almeida et al., 2010; Sakamaki et al., 2006; Wang, 2017). Interestingly, it has also been suggested that coupling of the cell cycle with metabolism is largely achieved by timely destruction of key tricarboxylic acid cycle enzymes, in a Skp2- and cyclin E/CDK2-dependent manner, and this regulatory connection has been implicated in prostate cancer (Liu et al., 2021). Therefore, there exists compelling evidence for links between two cancer hallmarks, aberrant cell cycle and addiction to glycolysis. Another metabolic event associated with rapid cellular proliferation is the high rate of lactate production from glucose, despite the availability of oxygen for mitochondrial respiration (Liberti and Locasale, 2016; Warburg, 1956; Koppenol et al., 2011). Lactate has also been shown to directly influence cell division via regulation of the anaphase-promoting complex (APC), which is crucial for metaphase to anaphase transition during mitosis (Liu et al., 2023). However, nothing is known about how lactate level affects a eukaryotic cell's proliferative activity. In this study, we report a systematic study of the effect of changing the carbohydrate source of the cells from glucose to lactate with respect to cell behavior, fate and also the regulation of major determinants of mitotic timing, namely cyclin-dependent kinase (CDK) and mitogen-activated protein kinase (MAPK).

We have used, for our studies, the unicellular yeast *Schizosaccharomyces pombe*, which is an excellent and established model system for studying cell division (Hedges, 2002). It has been reported earlier that there exists a strong correlation between CDK Cdc2 activity (Cdk1 homolog) and MAPK Sty1 (p38 homolog) activity, which regulates mitotic timing decisions in *S. pombe*. Cdc2 can be activated by the dephosphorylation of its Y15 residue by the Cdc25 phosphatase (Ducommun et al., 1990), and Sty1 is known to be able to negatively regulate Cdc25 (Paul et al., 2018). Moreover, aberrant Cdc2 activity leads to increased Sty1 phosphorylation (Paul et al., 2018), while inhibition of Cdc2 leads to decreased Sty1 phosphorylation (Ghosal et al., 2020) and the Wis1 MAPKK is a mediator of this communication (Ghosal et al., 2020). We also earlier reported that Sty1 is important for regulating functions of the APC (Basu et al., 2022). In this study, we report observations that show that the functional communication between Cdc2 and Sty1 activities is abolished in cells that have been temporarily shifted to medium containing lactate as the carbon source instead of glucose. We also show that activation of Sty1 actually leads to cell death in these conditions. Surprisingly, aberrant increase in Cdc2 activity delays cell division and promotes cell survival under these conditions and actually opposes the Sty1-dependent loss of viability of these cells. We also observed that these cell fate outcomes are associated with alterations in the metabolic state of the cell, including reactive oxygen species (ROS) levels, oxygen consumption rates (OCRs) and lactate dehydrogenase (LDH) activity. The increased LDH activity in cells with high intrinsic Cdc2 activity is an interesting finding, and further investigation of the molecular mechanism responsible for it and extrapolation of such findings could lead to better understanding of the biochemical events associated with tumor progression and metastasis.

Our observations shed significant light on how lactate interferes with and possibly reprograms canonical cell cycle regulation. Altered CDK activity and altered metabolism, especially in the presence of lactic acid, are simultaneously associated with neoplastic disorders, and hence these results are significant for understanding disease progression in such conditions in higher eukaryotes as well.

## RESULTS

### Sty1 activity is associated with accelerated mitotic entry and loss of cell viability in *S. pombe* cells shifted to medium containing lactic acid instead of glucose

**Fig. 1.**
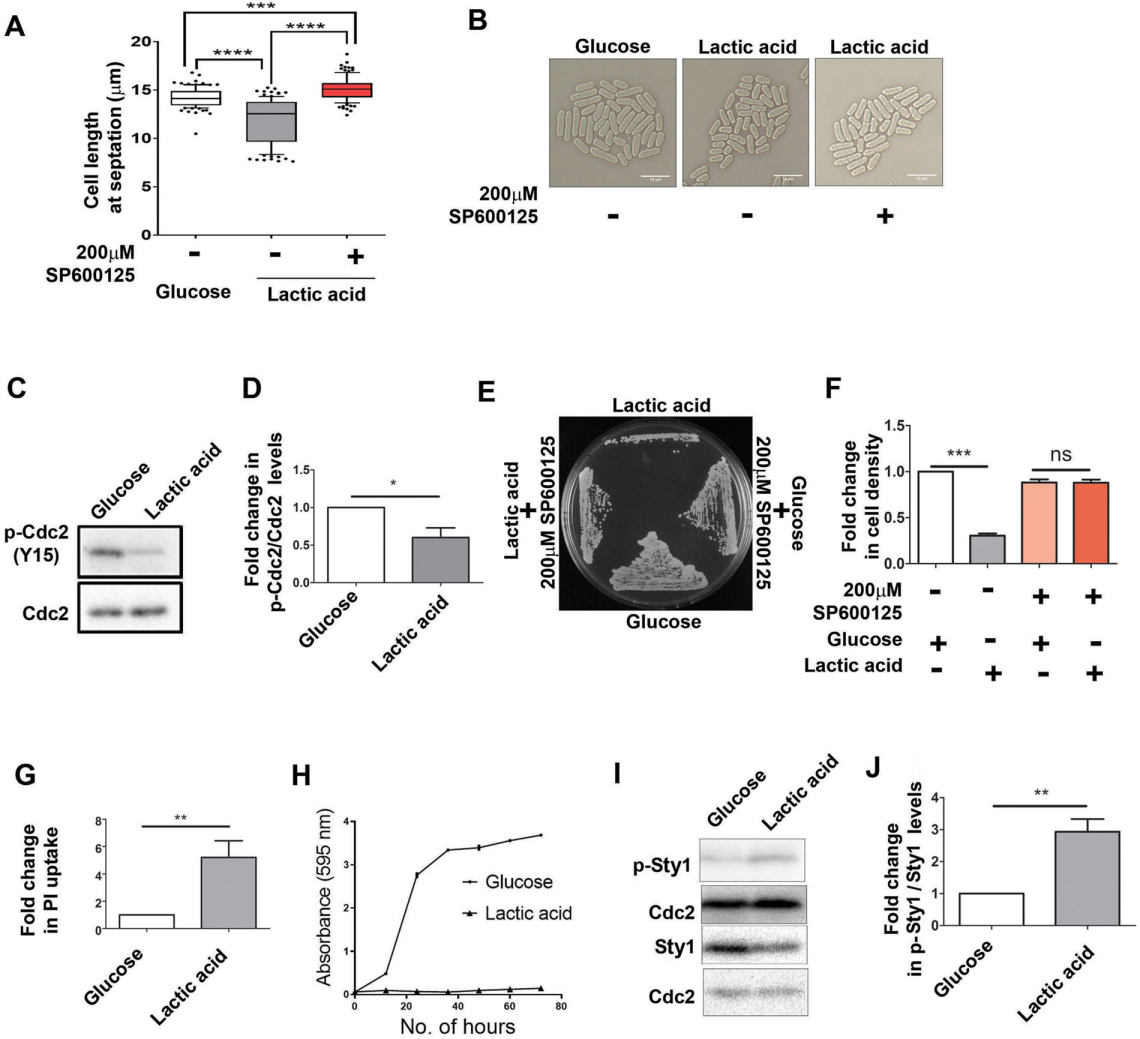
**Sty1 activation leads to decreased cell length at septation and increased cell death after glucose to lactate shift.** (A) Quantification of the cell length at septation of wt cells before and after treatment with Sty1 inhibitor (SP600125) in 2% glucose- or 2% lactate-containing medium for 3 h, using ImageJ software. The whiskers are drawn down to the 10th percentile and up to the 90th (*n*>100). A one-way ANOVA was done to compare the three groups and revealed a significant difference between their means [*F*(2304=108.7), *P*<0.0001]. Post hoc analysis using the Tukey's HSD test showed that the mean cell length at septation for cells growing in glucose was significantly different from that of those growing in lactate [****P<0.0001, 95% confidence interval (C.I.)=(1.714 to 2.751)] or in lactate+SP600125 [***P=0.0002, 95% C.I.=(−1.402 to −0.3722)] and that the mean cell length at septation for cells growing in lactate was significantly different from that of cells growing in lactate+SP600125 [****P<0.0001, 95% C.I.=(-3.632 to −2.607)]. (B) Brightfield images of wt cells before and after treatment with Sty1 inhibitor (SP600125) in 2% glucose- or 2% lactate-containing medium for 3 h. Scale bars: 10 μm. (C,D) Levels of phosphorylated-Cdc2 (Y15) in wt cells growing in 2% glucose- or 2% lactate-containing medium for 1 h were determined by immunoblotting (C) and quantified using ImageJ (D). *P<0.05; Cdc2 levels were used for normalization. (E) wt cells were grown to mid-log phase and then allowed to grow in 2% glucose- or 2% lactate-containing medium in the presence or absence of Sty1 inhibitor (SP600125) for 3 h, before being streaked on YES (2% glucose) medium. The plates were incubated at 30°C for 3 days before being photographed. (F) wt cells were grown to mid-log phase and then allowed to grow in 2% glucose- or 2% lactate-containing medium in the presence or absence of Sty1 inhibitor (SP600125) for 3 h. The extent of cell growth in liquid media was determined by recording optical density (OD) at 595 nm. ***P<0.001; ns, not significant. (G) Flow cytometric analysis of cell death (propidium iodide uptake) in wt cells in 2% glucose- or 2% lactate-containing medium for 3 h. **P<0.01. (H) Growth curve of cells growing in the presence of 2% glucose or lactic acid was followed by recording the absorbance of the cells at 595 nm at the indicated time intervals. (I,J) Levels of p-Sty1 and total Sty1 in wt cells growing in 2% glucose- or 2% lactate-containing medium for 1 h were determined by immunoblotting (I) and quantified using ImageJ (J). **P<0.01; Cdc2 levels were used for normalization. All data are representative of three independent experiments. All bar graphs represent mean±s.e.m. A one-tailed *t*-test was performed for evaluation of statistical significance, unless indicated otherwise.

In order to study the effect of alteration of carbon source from glucose to lactic acid, we shifted exponentially growing *S. pombe* cells from a medium containing 2% glucose to one containing 2% lactic acid for 3 h and looked at the changes in cell phenotype. We observed a decrease in the cell length at septation for these cells, indicating a possible acceleration of mitotic entry (Fig. 1A,B, Table 1). This indication was further supported by the fact that the inhibitory Tyrosine 15(Y15) phosphorylation of Cdc2 was found to be decreased in cells growing in the presence of lactic acid (Fig. 1C,D). The apparent change in mitotic timing, however, resulted in a loss of viability (Fig. 1E,G), which was also evident in the significantly reduced cell density compared with that in cells growing in the presence of glucose within 3 h of the shift to lactate-containing medium (Fig. 1F). To further confirm this possibility, we followed the growth of the *S. pombe* cells separately in both glucose- and lactate-containing media and observed that the cells failed to grow in a medium containing lactic acid (Fig. 1H). Mitotic timing as well as response to nutrient limitation in *S. pombe* has been previously linked with the MAPK pathway (Hartmuth and Petersen, 2009; Petersen and Nurse, 2007), so we next investigated the association of Sty1 activity with our observations. We found that Sty1 phosphorylation was high in these cells (Fig. 1I,J). We also found that inhibition of the MAPK Sty1 using the pharmacological inhibitor SP600125 abolished the cell length decrease seen upon the shift to lactic acid-containing medium (Fig. 1A, Table 1). Interestingly, Sty1 inhibition also rescued the growth rate (Fig. 1F) and viability defects of the wild-type (wt) cells growing in the presence of lactic acid (Fig. 1E). These results indicate that Sty1 activation inhibits the growth and proliferation of *S. pombe* cells in lactic acid-containing medium. We also found that inhibition of Sty1 prevented the accumulation of cells with shorter lengths at septation, indicating a prevention of any possible accelerated mitotic entry. The results indicated decreased cell length at septation, indicating possible mitotic acceleration, and cell death in the presence of lactic acid to be dependent on Sty1 activity. The fact that Sty1 inhibition allowed these cells to adapt to lactic acid as a carbon source also brings to light the possibility of a strong association between Sty1 activity and regulation of cellular metabolism.

**Table 1.**
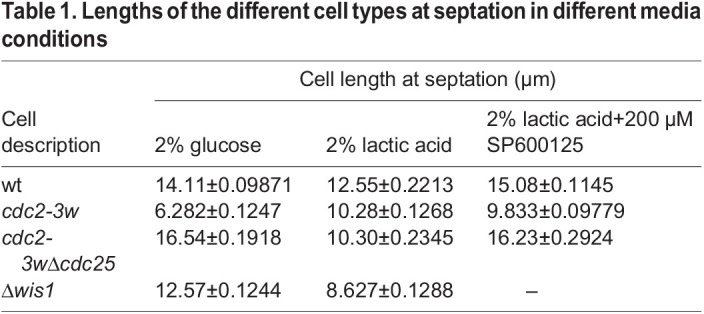
Lengths of the different cell types at septation in different media conditions

### Genetic modification leading to high intrinsic Cdc2 activity leads to better adaptation to lactic acid-containing medium

**Fig. 2.**
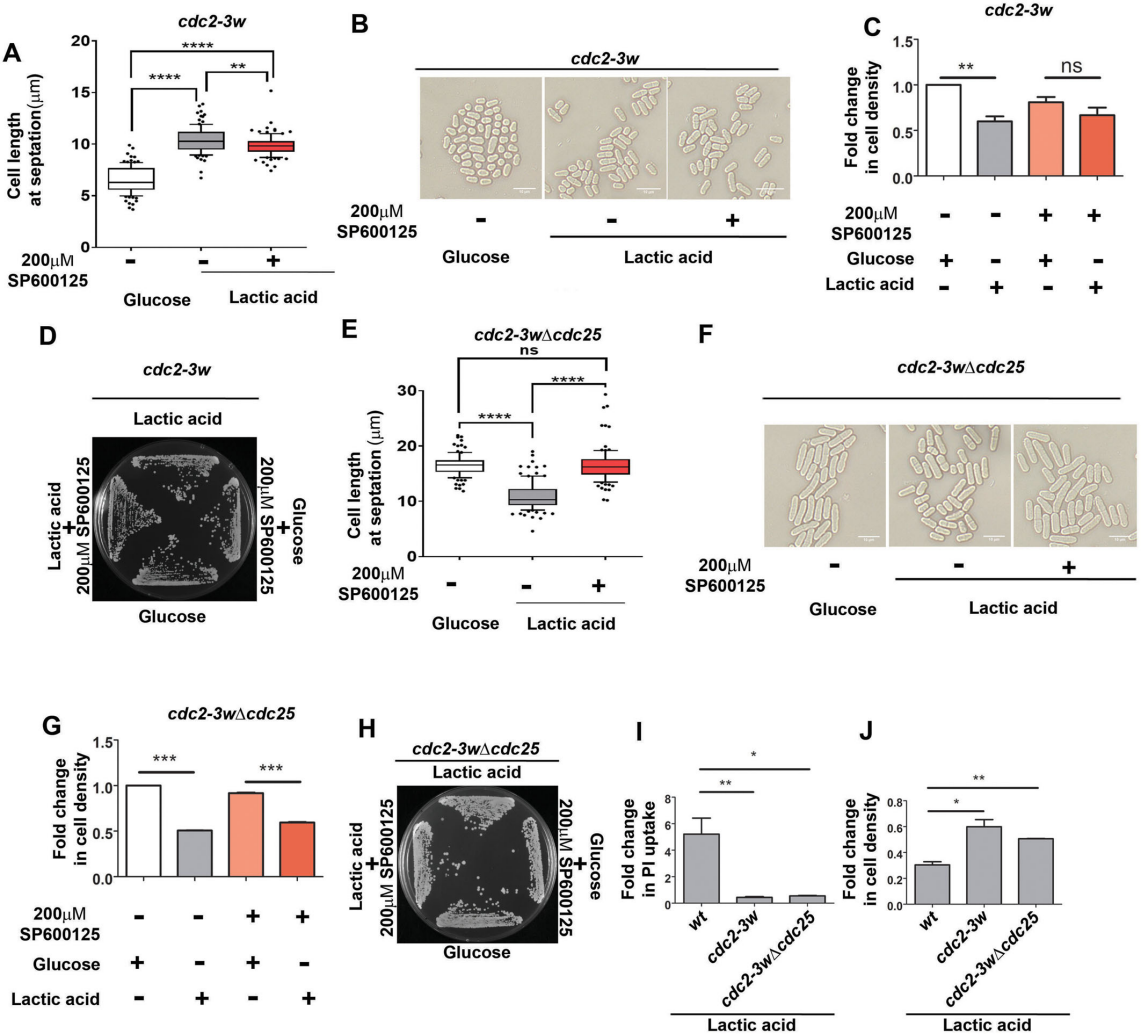
**CDK-dependent cell division decisions can be altered after glucose to lactate shift.** (A) Quantification of the cell length at septation of *cdc2-3w* cells in 2% glucose- or 2% lactate-containing medium for 3 h in the presence or absence of Sty1 inhibitor (SP600125), using ImageJ software. The whiskers are drawn down to the 10th percentile and up to the 90th (*n*>100). A one-way ANOVA was done to compare the three groups and revealed a significant difference between their means [*F*(2309=319.6), *P*<0.0001]. Post hoc analysis using the Tukey's HSD test showed that the mean cell length at septation for cells growing in glucose was significantly different from that of those growing in lactate [****P<0.0001, 95% C.I.=(−4.236 to −3.456)] or in lactate+SP600125 [****P<0.0001, 95% C.I.=(−3.705 to −2.927)] and that the mean cell length at septation for cells growing in lactate was significantly different from that of cells growing in lactate+SP600125 [**P=0.0047, 95% C.I.=(0.1366 to 0.9240)]. (B) Brightfield images of *cdc2-3w* cells growing in 2% glucose- or 2% lactate-containing medium for 3 h in the presence or absence of Sty1 inhibitor (SP600125). Scale bars: 10 μm. (C) *cdc2-3w* cells were grown to mid-log phase and then allowed to grow in 2% glucose- or 2% lactate-containing medium in the presence and absence of Sty1 inhibitor (SP600125) for 3 h. The extent of cell growth was determined by recording OD at 595 nm. **P<0.01; ns, not significant. (D) *cdc2-3w* cells were grown to mid-log phase and then allowed to grow in 2% glucose- or 2% lactate-containing medium in the presence and absence of Sty1 inhibitor (SP600125) for 3 h, and streaked on YES (2% glucose) medium. The plates were incubated at 30°C for 3 days before being photographed. (E) Quantification of the cell length at septation of *cdc2-3w*Δ*cdc25* cells before and after treatment with Sty1 inhibitor (SP600125) in the presence of glucose or lactic acid for 3 h, using ImageJ software. The whiskers are drawn down to the 10th percentile and up to the 90th (*n*>100). A one-way ANOVA was done to compare the three groups and revealed a significant difference between their means [*F*(2315=175.7), *P*<0.0001]. Post hoc analysis using the Tukey's HSD test showed that the mean cell length at septation for cells growing in glucose was significantly different from that of those growing in lactate [****P<0.0001, 95% C.I.=(4.742 to 6.369)] but not significantly different from that of cells growing in lactate+SP600125 [*P*>0.9999, 95% C.I.=(−0.8181 to 0.8238)]. However, the mean cell length at septation for cells growing in lactate was significantly different from that of cells growing in lactate+SP600125 [****P<0.0001, 95% C.I.=(−6.358 to −4.747)]. (F) Brightfield images of *cdc2-3w*Δ*cdc25* cells growing in 2% glucose- or 2% lactate-containing medium for 3 h in the presence or absence of Sty1 inhibitor (SP600125). Scale bars: 10 μm (G) *cdc2-3w*Δ*cdc25* cells were grown to mid-log phase and then allowed to grow in 2% glucose- or 2% lactate-containing medium in the presence and absence of Sty1 inhibitor (SP600125) for 3 h. The extent of cell growth was determined by recording OD at 595 nm. ***P<0.001. (H) *cdc2-3w*Δ*cdc25* cells were grown to mid-log phase and then allowed to grow in 2% glucose- or 2% lactate-containing medium in the presence and absence of Sty1 inhibitor (SP600125) for 3 h, and streaked on YES (2% glucose) medium. The plates were incubated at 30°C for 3 days before being photographed. (I) Flow cytometric analysis of cell death (propidium iodide uptake) in wt, *cdc2-3w* and *cdc2-3w*Δ*cdc25* cells growing in 2% lactate-containing medium for 3 h. **P<0.01; *P<0.05. (J) wt, *cdc2-3w* and *cdc2-3w*Δ*cdc25* cells were grown to mid-log phase and then allowed to grow in glucose or lactic acid for 3 h. The extent of cell growth was determined by recording OD at 595 nm. **P<0.01; *P<0.05. All data are representative of three independent experiments. All bar graphs represent mean±s.e.m. A one-tailed *t*-test was performed for evaluation of statistical significance, unless indicated otherwise.

Studies from our laboratory have earlier established a strong connection between Sty1 and Cdc2 activity during mitotic timing regulation in *S. pombe*. So to understand the molecular mechanism associated with the altered cellular behavior in the presence of lactic acid, we investigated the fate of *cdc2* mutants after glucose to lactate shift. We repeated our experiments in a *cdc2-3w* mutant (Basi and Enoch, 1996), which harbors a mutant allele of *cdc2* that is insensitive to Cdc25 and does not require Cdc25 for its activation. These cells also have increased Cdc2 activity and enter mitosis faster that wt cells and therefore have a decreased cell length at septation. When we shifted the *cdc2-3w* mutants from glucose- to lactate-containing medium for 3 h, we found a complete reversal of this phenotype and that this mutant exhibited increased cell length at septation, indicating that, in the presence of lactic acid, *S. pombe* cells with high Cdc2 activity experience a delay in mitotic initiation (Fig. 2A,B, Table 1). The *cdc2-3w* mutant did show a growth delay as well but to a lesser extent than wt cells. Surprisingly, these mutants remained viable even after glucose to lactate shift (Fig. 2C,D). Thus, the aberrantly high Cdc2 activity in these cells is associated with their ability to utilize lactic acid better than wt cells. It is to be noted here that wt cells in similar conditions also have high Cdc2 activity, although with entirely different outcomes both in terms of mitotic timing as well as viability. We repeated our experiment in the *cdc2-3wΔcdc25* cells. Since the mutant *cdc2-3w* allele does not require Cdc25 for its activation, the latter can be deleted in this background. Surprisingly, we found that these cells exhibit a similar phenotype as wt cells, i.e. reduced cell length at septation (Fig. 2E,F, Table 1). The surprising part of this observation is that deletion of *cdc25^+^* reversed the mitotic timing phenotype of *cdc2-3w* cells. The growth and viability of *cdc2-3wΔcdc25* cells, however, remained similar to those of the *cdc2-3w* mutants (Fig. 2G-J). These interesting observations implicate Cdc2, along with Sty1, as an important regulator of the cellular metabolic pathways. These results again reinforce the possibility that metabolic reprogramming of the cell can alter the outcome of CDK-dependent cell division decisions in *S. pombe*. Further investigations are, however, needed to understand the molecular mechanism of this extremely important layer of eukaryotic cell division control. It may be noted that although inhibition of Sty1 restored the cell length of septation of *cdc2-3wΔcdc25* cells to a similar length as seen in glucose-containing medium in the absence of the inhibitor (Fig. 2E, Table 1), a similar restoring effect was not seen when cell densities were compared (Fig. 2G). Further investigation is necessary to understand the subtle intricacies of the effect of glucose to lactate shift on this mutant.

One interesting phenomenon that these results however clearly establish is that the presence of the misregulated Cdc2 leading to high intrinsic Cdc2 activity is the major determinant of cellular survival after being shifted to lactic acid, as both *cdc2-3w* and *cdc2-3wΔcdc25* cells are viable after lactic acid shift, in sharp contrast to wt cells (Fig. 2I,J). These results therefore also indicate the gaps in the current understanding of the functional implications of the *cdc2-3w* mutation and the role of Cdc25 in cells bearing the *cdc2-3w* mutation.

### Extent of Sty1 phosphorylation and its functional interaction with Cdc2 and Cdc25 regulates mitotic timing decisions and cell survival upon glucose to lactate shift

**Fig. 3.**
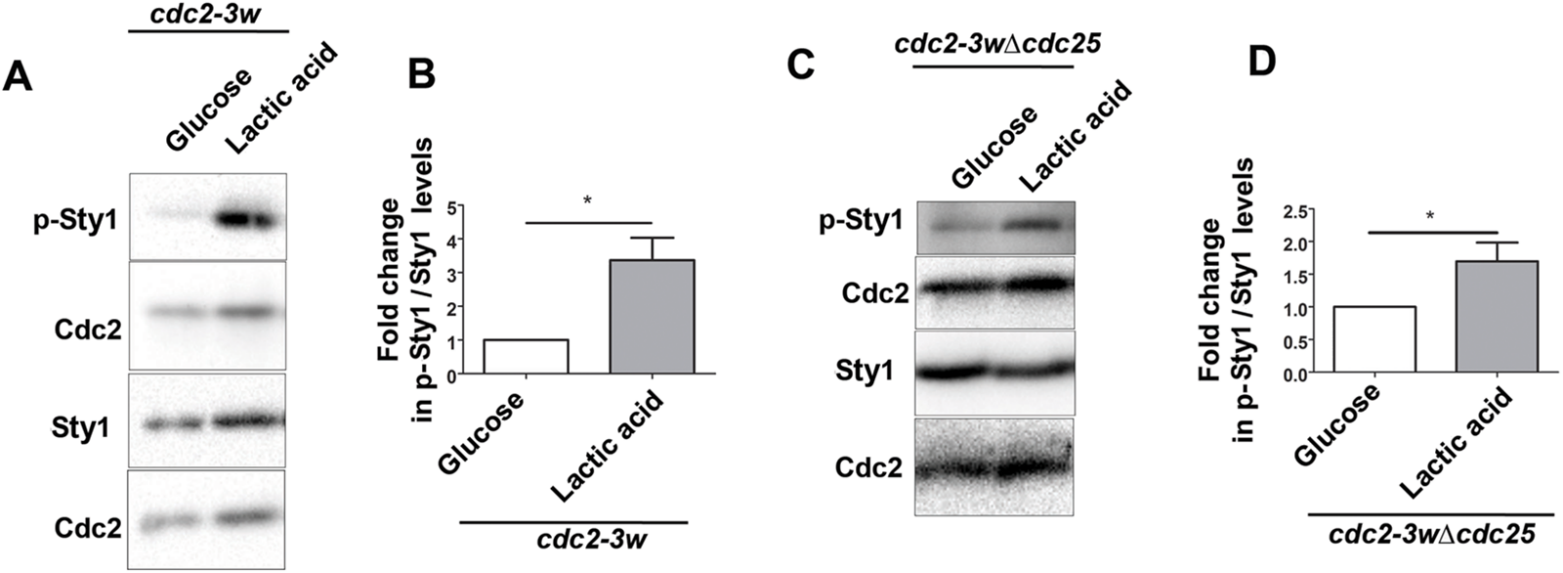
**Sty1 activity is associated with varied phenotypic outcomes after glucose to lactate shift.** (A,B) Levels of p-Sty1 and total Sty1 in *cdc2-3w* cells growing in 2% glucose- or 2% lactate-containing medium for 1 h were determined by immunoblotting (A) and quantified using ImageJ (B). *P<0.05; Cdc2 levels were used for normalization. (C,D) Levels of phosphorylated-Sty1 and Sty1 levels in *cdc2-3w*Δ*cdc25* cells growing in 2% glucose- or 2% lactate-containing medium for 1 h were determined by immunoblotting (C) and quantified using ImageJ (D). *P<0.05; Cdc2 levels were used for normalization. All data are representative of three independent experiments. All bar graphs represent mean±s.e.m. A one-tailed *t*-test was performed for evaluation of statistical significance.

A comparison of the phenotypes of the wt, *cdc2-*3w and *cdc2-3wΔcdc25* cells indicates that the *cdc2-3wΔcdc25* cells have an intermediate phenotype that is similar to that of wt cells in terms of mitotic timing and similar to that of *cdc2-3w* cells in terms of viability or ability to utilize lactic acid instead of glucose. We had earlier reported that the influence of Sty1 on mitotic timing in *S. pombe* cells has a dose-dependent effect, whereby moderate increase in Sty1 activity leads to mitotic acceleration, while a high increase in Sty1 activity leads to mitotic delay (Paul et al., 2018). We therefore decided to investigate whether the trends of altered cell fate upon glucose to lactate shift could be associated with any differences in Sty1 activity of these cells. Sty1 activity is known to be high in *cdc2-3w* cells compared with wt cells in normal conditions (Ghosal et al., 2020), and we found that lactate shift causes a higher increase in Sty1 phosphorylation in *cdc2-3w* cells (Fig. 3A,B) than in wt cells (Fig. 1I,J). But Sty1 activity in *cdc2-3wΔcdc25* cells (Fig. 3C,D) was lower than that in wt cells (Fig. 1I,J) under similar conditions. These results also indicate that loss of Cdc25 function can affect the extent of Sty1 activation in response to lactic acid shift. Taken together, these observations show that Cdc25-dependent alteration of Sty1 activity can together coordinate an outcome in light of glucose to lactate shift.

As mentioned earlier, inhibition of Sty1 reverses the mitotic acceleration phenotype of wt cells after glucose to lactate shift and also rescues their viability defect. We repeated these experiments in *cdc2-3w* cells and found that inhibition of Sty1 had no effect on the phenotypes (Fig. 2A,B) of the *cdc2-3w* mutant. In *cdc2-3wΔcdc25* cells, however, the inhibition of Sty1 led to reversal of the mitotic acceleration phonotype (Fig. 2E,F) just like in wt cells. The comparison of these phenotypes also suggests that even though Sty1 is activated, it is not able to accelerate mitotic entry in *cdc2-3w* cells in the presence of Cdc25.

### Communication between Cdc2 and Sty1 activity is altered after glucose to lactate shift

**Fig. 4.**
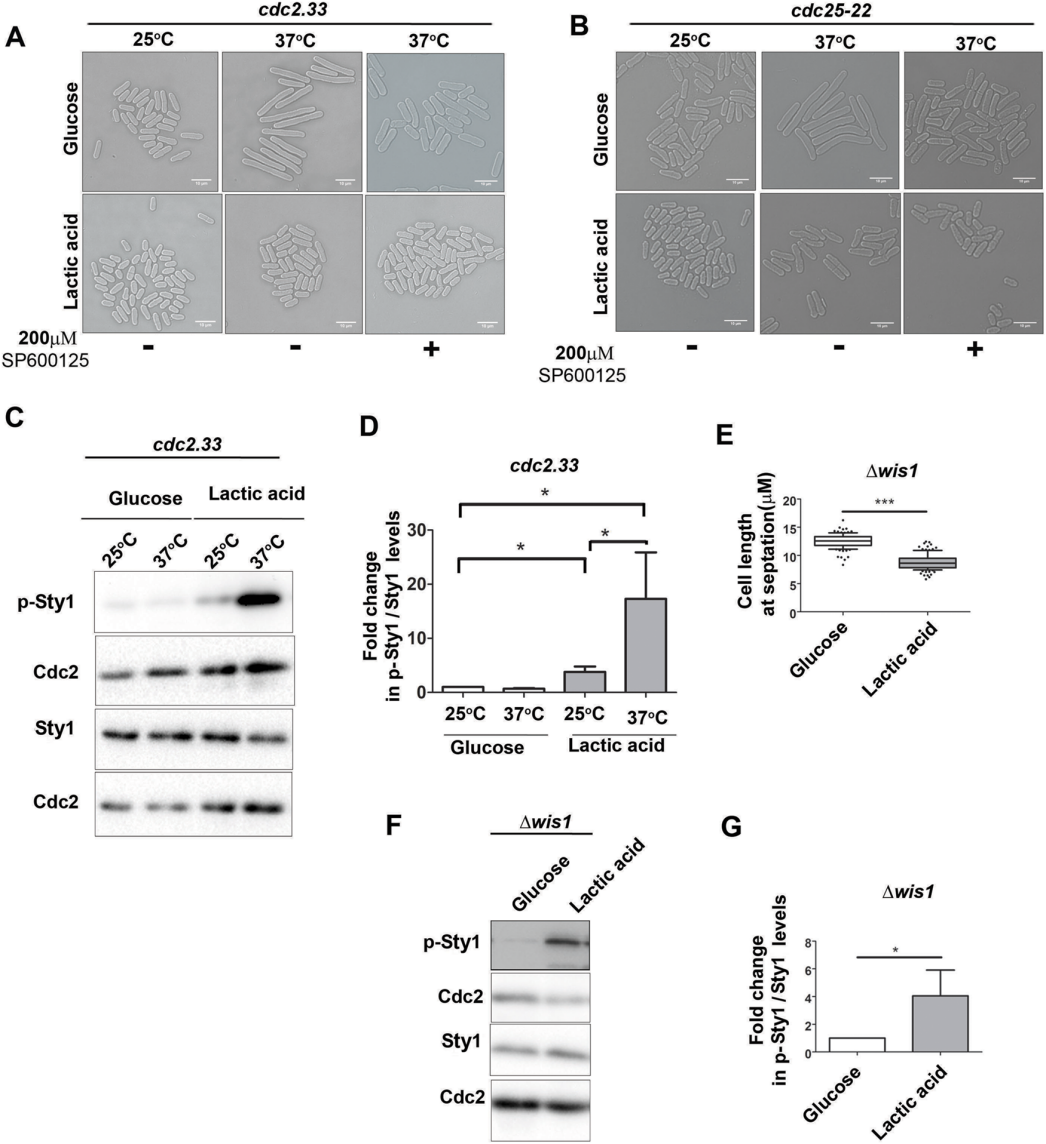
**The known correlation between Cdc2 activity and levels of phosphorylated Sty1 is lost after glucose to lactate shift.** (A) Brightfield images of *cdc2.33* cells growing at permissive (25°C) and non-permissive (37°C) temperatures in 2% glucose- or 2% lactate-containing medium in the presence and absence of Sty1 inhibitor (SP600125) in the presence of glucose or lactic acid for 4 h. Scale bars: 10 μm. (B) Brightfield images of *cdc25-22* cells growing at permissive (25°C) and non-permissive (37°C) temperatures in 2% glucose- or 2% lactate-containing medium for 4 h in the presence and absence of Sty1 inhibitor (SP600125). Scale bars: 10 μm. (C,D) Levels of phosphorylated-Sty1 and Sty1 levels in *cdc2.33* mutants growing at permissive (25°C) and non-permissive (37°C) temperatures in 2% glucose- or 2% lactate-containing medium for 4 h determined by immunoblotting (C) and quantified using ImageJ (D). *P<0.05; Cdc2 levels were used for normalization. (E) Quantification of the cell length at septation of Δ*wis1* cells growing in 2% glucose- or 2% lactate-containing medium for 3 h, using ImageJ software. The whiskers are drawn down to the 10th percentile and up to the 90th (*n*>100). ***P<0.001. (F,G) Levels of p-Sty1 and total Sty1 in Δ*wis1* cells growing in 2% glucose- or 2% lactate-containing medium for 1 h determined by immunoblotting (F) and quantified using ImageJ (G). *P<0.05; Cdc2 levels were used for normalization. All data are representative of three independent experiments. All bar graphs represent mean±s.e.m. A one-tailed *t*-test was performed for evaluation of statistical significance.

To further characterize the unexpected finding that glucose to lactate shift can lead to mitotic delay specifically in cells with high intrinsic Cdc2 activity, we decided to check the fate of loss-of-function mutants of Cdc2 and Cdc25 (positive regulator of Cdc2), respectively, during similar conditions. We took *cdc2.33* and *cdc25-22* mutant *S. pombe* cells. These cells harbor a temperature-sensitive allele of *cdc2* and *cdc25*, respectively. When incubated at the non-permissive temperature of 37°C, these cells become deficient in Cdc2 and Cdc25 activity, respectively, and are unable to initiate mitosis. Consequently, they appear significantly elongated, indicating G2/M arrest of *S. pombe* cells. These mutants were first grown at the permissive temperature of 25°C and then shifted to the non-permissive temperature for 4 h in glucose- and lactate-containing medium separately. While cells incubated in glucose-containing medium exhibited the expected elongated cell length phenotype (Fig. 4A,B), the cells in the lactate-containing medium showed no change in cell length at the non-permissive temperature (Fig. 4A,B). This indicated that these cells did not get arrested at the G2-M boundary. These experiments indicated that the G2/M arrest of *S. pombe* cells due to loss of Cdc2 or Cdc25 function was abolished when the cells were shifted to lactate-containing medium. Interestingly, *cdc2.33* and *cdc25-22* cells shifted to non-permissive temperature in glucose-containing medium in the presence of Sty1 inhibitor also exhibited the same phenotype as the cells incubated in lactate-containing medium in the absence of Sty1 inhibitor. Thus, addition of Sty1 inhibitor apparently had no effect on the phenotypes of the *cdc2.33* or *cdc25-22* cells after glucose to lactate shift (Fig. 4A,B). We earlier showed that decrease in Cdc2 activity is associated with decreased Sty1 phosphorylation and activity in *S. pombe* cells (Ghosal et al., 2020). Surprisingly, we found that, in lactate-containing medium, there was no decrease in Sty1 phosphorylation in *cdc2.33* cells when shifted from the permissive to non-permissive temperature, and in both cases the extent of Sty1 phosphorylation after glucose to lactate shift remained much high compared to that in the control cells growing in glucose-containing medium (Fig. 4C,D). So the correspondence between Cdc2 and Sty1 activity seems to be lost after glucose to lactate shift. The communication between Cdc2 and Sty1 is known to be mediated by the Sty1 regulatory MAPKK Wis1 **(**Ghosal et al., 2020**)**. We repeated our experiments in Δ*wis1* cells and found that they behave similarly to wt cells, i.e. there was a decrease in cell length at septation (Fig. 4E, Table 1). Deletion of *wis1^+^* did not abolish Sty1 phosphorylation (Fig. 4F,G) in these conditions, indicating that Sty1 activation during glucose to lactate shift occurs in a Wis1-independent manner. This lack of dependence of Sty1 on Wis1 during glucose to lactate shift might be one possible reason for the disruption of the communication between Sty1 and Cdc2 activity in *S. pombe* cells during glucose to lactate shift.

### The distinct metabolic states of wt and *cdc2-3w* cells influence the differences in their mitotic timing

**Fig. 5.**
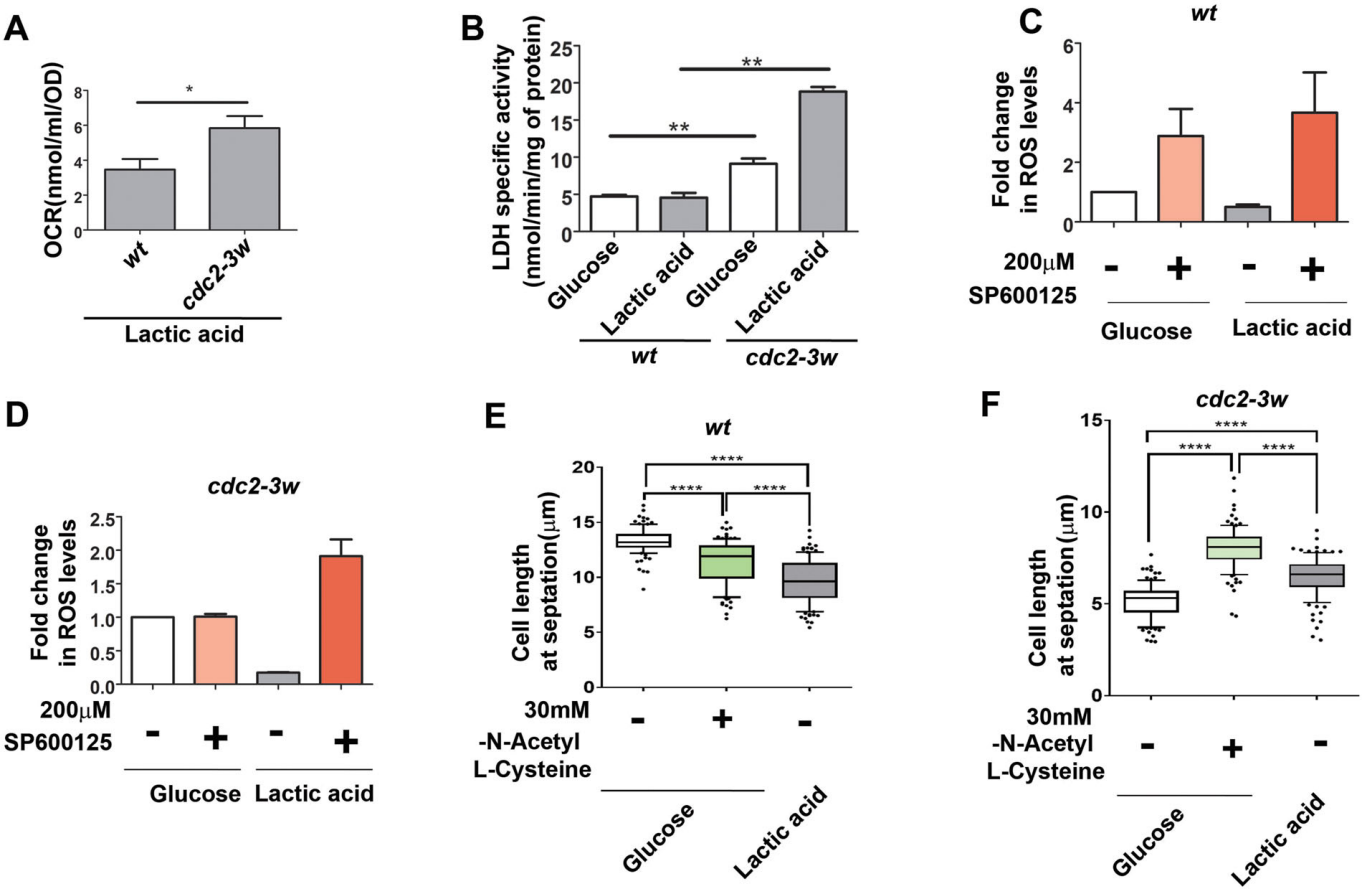
**Mitotic timing decisions and metabolic status of the cell are linked.** (A) wt and *cdc2-3w* cells were grown in 2% glucose medium to OD 0.8, and then either shifted to 2% lactate medium or continued to grow in glucose medium for another 3 h. Endogenous oxygen consumption rate (OCR) was measured using a Clark type O_2_ electrode at 30°C after suspending cells in fresh 2% glucose or 2% lactate medium at a concentration of 10^8^ cells/ml. *P<0.05. (B) Lactate dehydrogenase (LDH)-specific activity was measured spectrophotometrically at 340 nm in wt and *cdc2-3w* cells. **P<0.01. (C) Flow cytometric analysis of ROS generation after treatment with Sty1 inhibitor (SP600125) in wt cells growing in 2% glucose- or 2% lactate-containing medium for 1 h. (D) Flow cytometric analysis of ROS generation after treatment with Sty1 inhibitor (SP600125) in *cdc2-3w* cells growing in 2% glucose- or 2% lactate-containing medium for 1 h. (E) Quantification of the cell length at septation of wt cells growing in 2% glucose [in the presence and absence of 30 mM N-acetyl-L-cysteine (NAC)] or 2% lactate-containing medium for 3 h, using ImageJ software. The whiskers are drawn down to the 10th percentile and up to the 90th (*n*>100). A one-way ANOVA was done to compare the three groups and revealed a significant difference between their means [*F*(2312=107.4), *P*<0.0001]. Post hoc analysis using the Tukey's HSD test showed that the mean cell length at septation for cells growing in glucose was significantly different from that of those growing in glucose+NAC [****P<0.0001, 95% C.I.=(1.343 to 2.517)] or in lactate [****P<0.0001, 95% C.I.=(3.030 to 4.191)] and that the mean cell length at septation for cells growing in glucose+NAC was significantly different from that of cells growing in lactate [****P<0.0001, 95% C.I.=(1.107 to 2.253)]. (F) Quantification of the cell length at septation of *cdc2-3w* growing in 2% glucose (in the presence and absence of 30 mM NAC) or 2% lactate-containing medium for 3 h, using ImageJ software. The whiskers are drawn down to the 10th percentile and up to the 90th (*n*>100). A one-way ANOVA was done to compare the three groups revealed a significant difference between their means [*F*(2303=190), *P*<0.0001]. Post hoc analysis using the Tukey's HSD test showed that the mean cell length at septation for cells growing in glucose was significantly different from that of those growing in glucose+NAC [****P<0.0001, 95% C.I.=(−3.277 to −2.570)] or in lactate [****P<0.0001, 95% C.I.=(−1.713 to −1.014)] and that the mean cell length at septation for cells growing in glucose+NAC was significantly different from that of cells growing in lactate [****P<0.0001, 95% C.I.=(1.208 to 1.911)]. All data are representative of three independent experiments. All bar graphs represent mean±s.e.m. A one-tailed *t*-test was performed for evaluation of statistical significance, unless indicated otherwise.

Metabolic differences between cell utilizing glucose or lactate are expected to be associated with the cellular respiration of the cells. To assess the same in our experimental conditions, we checked the OCR in wt cells and *cdc2-3w* mutants. We found that the *cdc2-3w* mutants showed a much better OCR in lactate-containing medium than wt cells (Fig. 5A). These results indicate that mitochondrial function in *cdc2-3w* cells in the presence of lactate is better than that of the *wt* cells in similar conditions. This is in accordance with the earlier observation that *cdc2-3w* cells are more viable in these conditions compared with wt cells (Fig. 1E, Fig. 2D). These results, in addition to the earlier observations regarding the better adaptation of the *cdc2-3w* cells to lactate-containing medium, prompted us to investigate whether the *cdc2-3w* mutant was more efficient at metabolizing lactate. In order to understand that, we estimated the activity of LDH in both wt and *cdc2-3w* cells. Indeed, we found that the LDH activity in *cdc2-3w* mutants was almost 4-fold higher than that in wt cells (Fig. 5B). These results indicate that the survival of *S. pombe* cells in lactate-containing medium is associated with increased LDH activity and that this increase is absent in wt cells. Further studies are, however, required to understand the molecular mechanism responsible for the increased LDH activity of the *cdc2-3w* cells. We also measured the ROS levels of wt cells and *cdc2-3w* cells growing in glucose- or lactate-containing medium and found that ROS levels are reduced upon glucose to lactate shift in wt cells (Fig. 5C) and *cdc2-3w* (Fig. 5D) mutants. We treated these cells with the Sty1 inhibitor and found that inhibition of Sty1 leads to increased ROS levels in wt cells under both conditions. In *cdc2-3w* cells, however, this increase is less than that of wt cells in lactate-containing medium, and in glucose-containing medium there is hardly any increase in the ROS levels of *cdc2-3w* cells after inhibition of Sty1 (Fig. 5C,D). Thus, the ability of Sty1 in promoting ROS-scavenging mechanisms (Hagihara et al., 2014) appears to be is much less pronounced in the *cdc2-3w* cells. Since we found ROS levels to be lower after glucose to lactate shift, we decided to check the association of this phenotype with the mitotic timing phenotypes we observed. To mimic the lowering in ROS levels, we treated wt and *cdc2-3w* cells with N-acetyl-L-cysteine, a well-known ROS-scavenging agent. We found that treatment with N-acetyl-L-cysteine resulted in similar cell length phenotypes as in the glucose to lactate shift conditions. The cell length at septation for wt cells decreased (Fig. 5E) while that of *cdc2-3w* cells increased (Fig. 5F). These results indicate that the mitotic timing of *S. pombe* cells can be dictated by their ROS levels and that aberrant Cdc2 activity under such conditions can result in a completely contrasting outcome. It is therefore also possible that the altered cellular fates in glucose- or lactate-containing medium might be due to the difference in intracellular ROS levels. It would be interesting to explore whether other metabolic changes leading to altered redox status of the cell can also influence the mechanism of the mitotic timing regulation of these cells.

## DISCUSSION

Classically, Cdc2 activity is known to have a direct correlation with mitotic timing. Mitotic entry is facilitated by an increase in Cdc2 activity resulting from dephosphorylation of its Y15 residue, while decreased Cdc2 activity is known to cause a delay in mitotic initiation. Cdc2 mutants like *cdc2-3w* spontaneously lose this dephosphorylation, and therefore Cdc2 is activity is intrinsically high in these cells and they initiate mitosis faster than wt cells, which results in them having a shorter cell length at septation. In this study, we present evidence that the association between mitotic timing and Cdc2 activity is strongly influenced by the metabolic status of the cell and that intrinsic aberrations in Cdc2 activity completely dictate cell fate outcome during metabolic perturbations. Our results show that changing the nutrient source from glucose to lactate, resulting in decreased intracellular ROS level, can cause a mitotic delay in *cdc2-3w* mutants in spite of the intrinsically high Cdc2 activity. Surprisingly, this delay was seen only in these mutants, while in wt cells the correlation between Cdc2 activity and cell length remained unaltered. More significantly, while wt cells fail to proliferate under such conditions and are inviable, the *cdc2-3w* mutants adapt well to the altered metabolic conditions and retain viability. We also found that the loss of viability of *wt* cells was dependent on Sty1 activity as inhibiting Sty1 rescued the viability of *wt* cells after glucose to lactate shift. This rescue was concomitant with restoration of their cell lengths to normal values, indicating that the possible mitotic timing aberrations were also prevented. Interestingly, we found that the response of *cdc2-3wΔcdc25* in terms of changes in cell length resulting from glucose to lactate shift both in the presence and absence of Sty1 inhibitor was similar to that of wt cells. This is further surprising because this implicates Cdc25 as being responsible for the delayed mitotic entry phenotype seen in the *cdc2-3w* mutant. This is also contrary to the known role of Cdc25 as a positive driver of mitosis. Moreover, the fact that while *cdc2-3w* cells did not respond to Sty1 inhibition but *cdc2-3wΔcdc25* cells did indicates that, in the presence of a hyperactive Cdc2, Sty1 and Cdc25 oppose each other's functions in terms of mitotic timing regulation. In terms of cell viability, however, the *cdc2-3wΔcdc25* had a similar fate as the *cdc2-3w* mutants as both the mutants were viable in these conditions. Taken together, these observations indicate that Sty1, Cdc2 and Cdc25 functions are simultaneously associated with the regulation of mitotic timing in *S. pombe* cells shifted to lactic acid and that functional cross-talks between these molecules determine the outcome. The nature of these cross-talks, however, appear to be distinctly different from those reported in cells growing in presence of glucose, thus indicating a possibility of metabolic regulation of Sty1-Cdc25 and Sty1-Cdc2 functional relationships. Moreover, the alteration of these relationships had a direct effect on cellular metabolism as our observations also indicated that that the ROS-scavenging mechanisms dependent on Sty1 may be less effective in a *cdc2-3w* mutant.

We also probed the possible reason for better survival of the *cdc2-3w* mutants after glucose to lactate shift and found that these cells have a better OCR than wt cells when grown in the presence of lactate. A striking observation was that the LDH activity of the *cdc2-3w* mutant was much higher than that of the wt cells. This explains why these cells adapt better to the glucose to lactate shift. It will be interesting to investigate the mechanism responsible for increased LDH activity in *cdc2-3w* cells. The association between high intrinsic Cdc2 activity and increased LDH activity is a novel finding that we report. This result is very important due to the fact that LDH is an important marker for tumorigenesis and is also being explored as a target for cancer therapy (Feng et al., 2018). Some cancer-associated mutations make it possible for cancer cells to acquire and metabolize nutrients in a way conducive to proliferation rather than effective ATP production (Vander Heiden et al., 2009). Our observation that the *cdc2-3w* mutation associated with accelerated cell division in the unicellular eukaryote *S. pombe* also leads to metabolic adaptation leading to better proliferation in non-optimal conditions (lactate instead of glucose) therefore highlights the fundamental nature of such associations. Further investigations could potentially reveal significant aspects of the mechanism regulating the metabolic changes that promote survival of malignant cells.

Interestingly, we found that ROS quenching in *S. pombe* cells through addition of N-acetyl-L-cysteine led to the same kind of contrasting cell length changes in both wt and *cdc2-3w* as seen after glucose to lactate shift. This observation indicates that not only an alteration of the nutrient source but a change in intracellular ROS levels can also alter the mitotic timing of *S. pombe* cells. It may be noted here that previous studies have also indicated that ROS-dependent mechanisms can contribute significantly towards the regulation of important cell cycle regulatory phosphatases and the ubiquitin ligases including the activity of the APC. These mechanisms control major cell cycle phase transition-related events including cyclin level regulation. (Menon and Goswami, 2007; Lim et al., 2015; Havens et al., 2006). It would be interesting to explore the mechanism by which ROS levels dictate mitotic fate in the experimental conditions mentioned in this study and how aberrant Cdc2 activity can influence such mechanisms.

It appears quite logical that the alterations to the metabolic state of the cell can change the cell cycle progression and survival. However, the interesting and novel aspect of the observations reported in this study is the fact that altered cellular metabolism can not only change but actually reverse the functional outcome of mutations in important cell division regulators. Our results highlight that metabolic regulation of CDK activity and cell division may be mediated by additional mechanisms, which are still not well understood. CDKs are significantly associated with development of malignancies and play critical functions in the regulation of cell cycle transition. A complex regulatory network of genetic and epigenetic pathways regulate CDK activity, but this network becomes dysregulated as cancer develops. Our study indicates that there is much more left to be explored about the biochemical factors that can regulate CDK function. Further research into the role and underlying mechanisms of CDKs in the presence of complex metabolic cues will, as brought to light in this study, pave the way for better CDK-targeting cancer therapeutics in the future.

## MATERIALS AND METHODS

### Fission yeast strains, media and growth conditions

The *Schizosaccharomyces pombe* strains used are listed in Table 2. The cells were grown as described by Moreno et al. (1991). They were grown at 30°C (unless otherwise indicated) in yeast extract with supplements (YES) medium. The MAPK inhibitor SP600125 (Sigma-Aldrich, Bangalore, Karnataka, India) at a concentration of 200 µM was used for the inhibition of Sty1 activity (Hartmuth and Petersen, 2009). ROS-scavenging agent N-acetyl-L-cysteine (SRL, 47866) at a concentration of 30 mM was used as an antioxidant for the phenotypic studies.

**Table 2.**
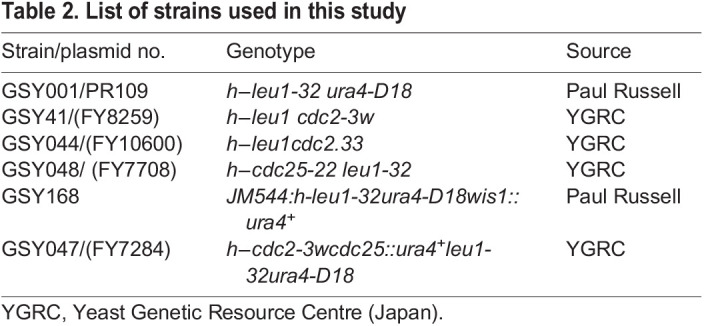
List of strains used in this study

#### Glucose to lactate shift

For glucose to lactate shift experiments, YES medium supplemented with 2% lactate instead of 2% glucose was used (pH adjusted to 5). The cells were first cultured up to mid-log phase in medium containing 2% glucose, harvested, washed and resuspended in medium containing 2% lactate and grown as indicated. For all biochemical assays, the cells continued to grow in the presence of 2% lactate for 1 h . For growth and viability assays and cell length at septation measurements, the growth was continued for 3 h in order to allow the cells to complete one cell division cycle at least so that the effects on cell division could be better understood. For OCR measurements also, the cells were allowed to grow in the presence of 2% lactate for 3 h to get a better idea about altered respiration rates.

### Microscopy

*S. pombe* cells were grown as indicated and live cells were examined using Olympus BX53 Microscope at 40× magnification unless mentioned otherwise. Brightfield images were taken using unstained cells. All images were taken and processed with the use of identical parameters. Cell length analysis was done using ImageJ software (http://imagej.nih.gov/ij/). At least 100 cells (*n*>100) were analyzed for the quantifications.

### Statistical analysis

**S**tatistical analysis of all quantitative data was performed using GraphPad Prism version 8.0.0 for Windows (www.graphpad.com). All bar graphs represent mean±s.e.m. For evaluation of statistical significance of all quantitative data, a one-way ANOVA with post hoc analysis was done when comparing more than two groups of data or a one-tailed *t*-test was performed when comparing two groups of data, and the *P*-values were used to estimate the significance of the results: ****P<0.0001; ***P<0.001; **P<0.01; *P<0.05; ns, not significant.

### Protein extract preparation

#### Cell lysate preparation for LDH assay

Cells were grown as indicated, cultured up to the mid-log phase in the presence of 2% glucose, normalized and then shifted to 2% lactic acid-containing medium for 1 h at 30°C. Cells were harvested and resuspended in the minimum volume of buffer containing 20 mM Tris-HCl, pH 7.5, 1 mM PMSF, 1× Protease Inhibitor Cocktail (Fermentas Life Sciences, R1321) followed by addition of glass beads. It was then vortexed for five cycles of 1 min each followed by 1 min of cooling on ice. Cell suspension was centrifuged at 12,000 ***g*** for 15 min at 4°C. The resulting supernatant was used as a cell-free cytosolic lysate for performing enzymatic assays (Mukherjee et al., 2023). Protein concentration was measured by the Bradford protein assay based on an absorbance shift of the Coomassie Brilliant Blue dye (Thermo Fisher Scientific, 1856209).

#### Denatured cell extracts for immunoblotting

Cell extracts were prepared under denaturing conditions. Harvested cells were resuspended in 20% trichloroacetic acid followed by addition of glass beads and vortexed at maximum speed for five 1 min pulses. The lysate was then transferred to a fresh microcentrifuge tube and then centrifuged at 14,170 ***g*** for 15 min. The supernatant was discarded and the pellets were washed with 70% ethanol. All steps were performed at 4°C and samples were kept on ice. Finally, the pellets were resuspended in Laemmli buffer and boiled for 5 min at 95°C.

### LDH activity assay

Using the cell-free lysates, cytosolic LDH (L-lactate:NAD+ oxidoreductase, EC 1.1.1.27) enzymatic activity was assayed by following the oxidation of NADH at 340 nm as a function of time as described previously (Place and Powers, 1984). Briefly, the LDH activity was assayed in 1 ml [sodium phosphate buffer (0.1 M, pH 7.5), 1 mM sodium pyruvate (Sigma-Aldrich, P2256) and 0.17 mM NADH (SRL, 77268)]. Then the commencement of the reaction was followed by the addition of 150 μg of cell lysate. The depletion of NADH was recorded for 5 min at 340 nm using a UV–Vis Spectrophotometer (Hitachi). The absorption coefficient used for NADH was 6220 M^−1^cm^−1^. The reaction rate without the addition of cell lysate was subtracted for the calculation of the specific activity in each set.

### Immunoblotting

The samples were then loaded onto 10% SDS–polyacrylamide gels. After transferring onto PVDF membranes, immunoblotting was performed using anti-Cdc2 (Santa Cruz Biotechnology, sc-53217) antibody, anti-phospho Cdc2 p34 (Tyr 15) (Santa Cruz Biotechnology, sc-7989) antibody, anti-phospho p38 [Cell Signaling Technology, 9211S; for phospho (p)-Sty1] or anti-Sty1 (lab stock) antibodies at 1:1000 dilution (or 1:2500 dilution for anti-Sty1). Immunoblots were developed using AdvanstaWesternBright™ ECL reagent (K-12045-D50).

### Protein level quantification from immunoblots

The signals from immunoblots were quantified using ImageJ. p-Sty1 and Sty1 levels were individually normalized using Cdc2 as a loading control. The ratio of the normalized intensity values of p-Sty1 and total Sty1, and the fold change of p-Sty1/Sty1 ratios with respect to the control cells, was calculated. pY15 Cdc2 levels were also normalized using Cdc2 signals.

### Viability assays

Cells were grown as indicated cultured up to the mid-log phase and then shifted to 2% lactic acid-containing medium for 3 h at 30°C. A loopful of cells from each sample was streaked on 2% glucose-containing YES agar plates and incubated at 30°C. The plates were photographed after 72 h.

For quantitative assays, cells grown under the indicated conditions were stained with propidium iodide (2 μg/ml). The proportion of dead cells was determined by quantifying the proportion of cells showing propidium iodide uptake by flow cytometry using a BD Accuri™ C6 Plus flow cytometer.

### Cellular OCR measurements

Cells were grown as indicated, cultured up to the mid-log phase in the presence of 2% glucose, normalized and then shifted to 2% lactic acid-containing medium for 3 h at 30°C. After incubation cells were centrifuged at 5000 ***g*** for 10 min, and cell pellets were suspended either in 2% glucose or 2% lactate medium at a concentration of 10^8^ cells/ml. Cellular OCR was measured using an Oxytherm fitted with a Clark electrode (Hansatech, UK). Respiration was measured for 4 min and then blocked with the CIV-specific inhibitor, sodium azide, at 1 mM concentration.

### ROS measurements

Cells were grown as indicated, cultured up to the mid-log phase in the presence of 2% glucose, normalized and then shifted to 2% lactic acid-containing medium for 1 h at 30°C. The cells were then incubated with 10 µM H_2_-DCFDA (Sigma-Aldrich, D6883-50MG) for 30 min, and the proportion of cells with ROS was ascertained by quantifying the number of cells showing DCF fluorescence by flow cytometry using the BD Accuri™ C6 Plus flow cytometer.
